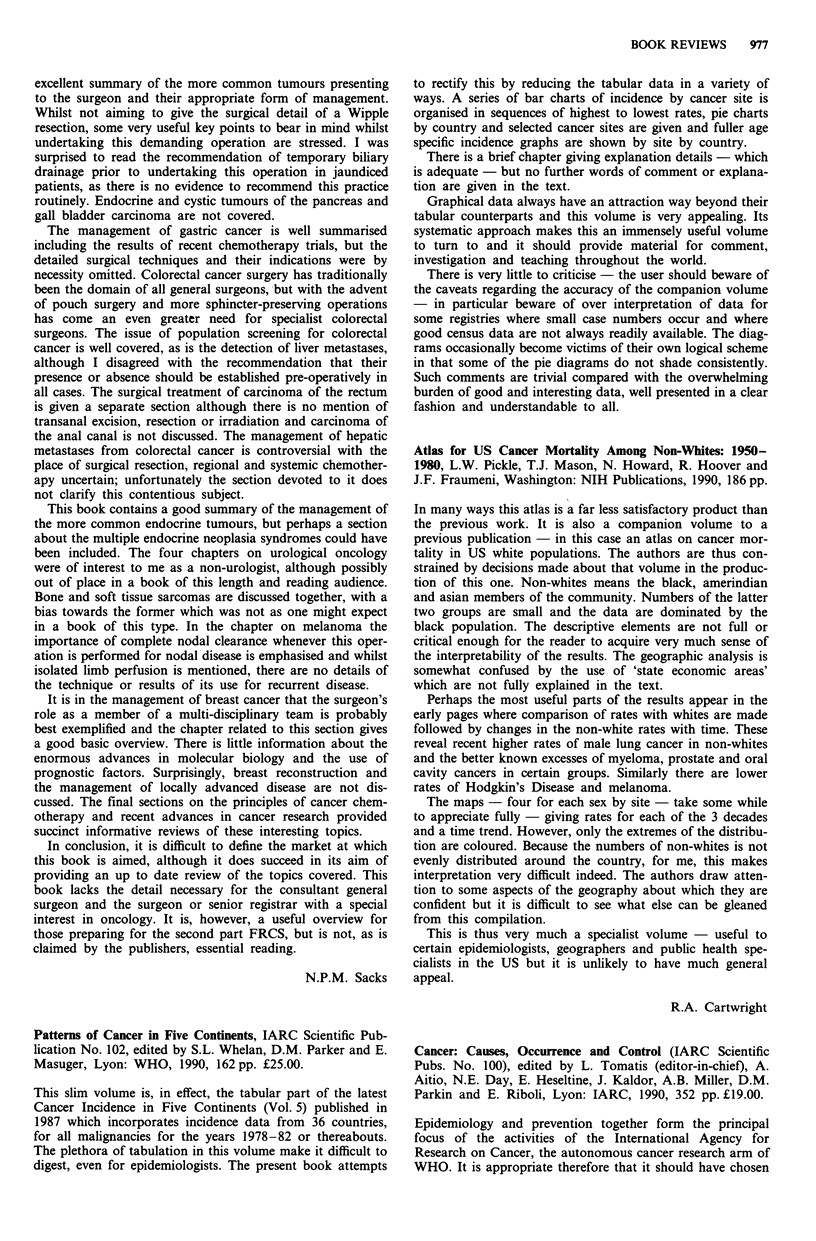# Patterns of Cancer in Five Continents

**Published:** 1991-11

**Authors:** 


**Patterns of Cancer in Five Continents**, IARC Scientific Publication No. 102, edited by S.L. Whelan, D.M. Parker and E. Masuger, Lyon: WHO, 1990, 162 pp. £25.00.

This slim volume is, in effect, the tabular part of the latest Cancer Incidence in Five Continents (Vol. 5) published in 1987 which incorporates incidence data from 36 countries, for all malignancies for the years 1978–82 or thereabouts. The plethora of tabulation in this volume make it difficult to digest, even for epidemiologists. The present book attempts to rectify this by reducing the tabular data in a variety of ways. A series of bar charts of incidence by cancer site is organised in sequences of highest to lowest rates, pie charts by country and selected cancer sites are given and fuller age specific incidence graphs are shown by site by country.

There is a brief chapter giving explanation details — which is adequate — but no further words of comment or explanation are given in the text.

Graphical data always have an attraction way beyond their tabular counterparts and this volume is very appealing. Its systematic approach makes this an immensely useful volume to turn to and it should provide material for comment, investigation and teaching throughout the world.

There is very little to criticise — the user should beware of the caveats regarding the accuracy of the companion volume — in particular beware of over interpretation of data for some registries where small case numbers occur and where good census data are not always readily available. The diagrams occasionally become victims of their own logical scheme in that some of the pie diagrams do not shade consistently. Such comments are trivial compared with the overwhelming burden of good and interesting data, well presented in a clear fashion and understandable to all.